# Correlation between heart rate variability and perioperative neurocognitive disorders in patients undergoing non-cardiac surgery: A retrospective cohort study

**DOI:** 10.1371/journal.pone.0297337

**Published:** 2024-04-02

**Authors:** Xiaoye Liu, Hengjun Wan, Huide Wang, GuanPeng Zhang, Qing Zhong, Xiaoxia Duan

**Affiliations:** 1 Department of Anesthesiology, The Affiliated Hospital, Southwest Medical University, Luzhou, Sichuan Province, China; 2 Anesthesiology and Critical Care Medicine Key Laboratory of Luzhou, Southwest Medical University, Luzhou, Sichuan Province, China; 3 Department of Anesthesiology, The People’s Hospital of Jianyang, Jianyang, Sichuan Province, China; 4 Department of Electrocardiogram, The Affiliated Hospital, Southwest Medical University, Luzhou, Sichuan Province, China; Chiba Daigaku, JAPAN

## Abstract

**Objective:**

With the improvement of medical level, the number of elderly patients is increasing, and the postoperative outcome of the patients cannot be ignored. However, there have been no studies on the relationship between preoperative heart rate variability (HRV) and Perioperative Neurocognitive Disorders (PND). The purpose of this study was to explore the correlation between (HRV) and (PND), postoperative intensive care unit (ICU), and hospital stay in patients undergoing non-cardiac surgery.

**Method:**

This retrospective analysis included 687 inpatients who underwent 24-hour dynamic electrocardiogram examination in our six departments from January 2021 to January 2022. Patients were divided into two groups based on heart rate variability (HRV): high and low. Possible risk factors of perioperative outcomes were screened using univariate analysis, and risk factors were included in multivariate logistic regression to screen for independent risk factors. The subgroup analysis was carried out to evaluate the robustness of the results. The nomogram of PND multi-factor logistic prediction model was constructed. The receiver operating characteristic (ROC) curve was drawn, and the calibration curve was drawn by bootstrap resampling 1000 times for internal verification to evaluate the prediction ability of nomogram.

**Result:**

A total of 687 eligible patients were included. The incidence of low HRV was 36.7% and the incidence of PND was 7.6%. The incidence of PND in the low HRV group was higher than that in the high HRV group (11.8% vs 5.2%), the postoperative ICU transfer rate was higher (15.9% than 9.3%P = 0.009), and the hospital stay was longer [15 (11, 19) vs (13), 0.015]. The multivariable logistic regression analysis showed that after adjusting for other factors, decreased low HRV was identified as an independent risk factor for the occurrence of PND (Adjusted Odds Ratio = 2.095; 95% Confidence Interval: 1.160–3.784; P = 0.014) and postoperative ICU admission (Adjusted Odds Ratio = 1.925; 95% Confidence Interval: 1.128–3.286; P = 0.016). This study drew a nomogram column chart for a multivariate logistic regression model, incorporating age and HRV. The calibration curve shows that the predicted value of the model for the occurrence of cardio-cerebrovascular events is in good agreement with the actual observed value, with C-index of 0.696 (95% CI: 0.626 ~ 0.766). Subgroup analysis showed that low HRV was an independent risk factor for PND in patients with gastrointestinal surgery and ASA Ⅲ, aged ≥ 65 years.

**Conclusion:**

In patients undergoing non-cardiac surgery, the low HRV was an independent risk factor for PND and postoperative transfer to the ICU, and the hospitalization time of patients with low HRV was prolonged. Through establishing a risk prediction model for the occurrence of PND, high-risk patients can be identified during the perioperative period for early intervention.

## Introduction

With the current state of an aging population, approximately one-third of elective surgeries are performed on patients aged >65 years [1]. Older patients often have chronic diseases, and 70% have a preoperative American Society of Anesthesiologists (ASA) classification of grade III-IV [2]. The incidence of postoperative complications, morbidity, mortality, and disability cannot be ignored. Preoperative examinations are often conducted to comprehensively evaluate patients, including an electrocardiogram (ECG), to reduce the risk of perioperative adverse events [3]. Perioperative ECG examination can predict or reveal possible cardiovascular risks, but the value of preoperative ECG examination remains controversial [4]. A 24-hour dynamic electrocardiography (DCG) can continuously record and analyze ECG changes of the human heart in an active and quiet state over 24 hours and analyze the heart rate variability (HRV) during day and night. Low HRV is linked to unfavorable outcomes in several illnesses, including hypertension, myocardial infarction, and diabetes [5]. There is a correlation between HRV and cognition [6]. In addition, abnormal perioperative HRV in patients undergoing orthopedic surgery is related to postoperative delirium, postoperative transfer to the intensive care unit (ICU), and extended hospital stay [7, 8]. However, research on this topic is lacking. This study aimed to investigate the correlation between 24-hour DCG abnormality and perioperative adverse outcomes, screen for risk factors of perioperative adverse outcomes, establish predictive models and detect the predictive values, and provide a reference for preoperative evaluation of patients through the analysis of clinical data of patients.

## Materials and methods

### Ethics statement

This study was registered in the Chinese Clinical Trial Center (http://www.chictr.org.cn) with registration number ChiCTR2300074890. This study was conducted in accordance with the Declaration of Helsinki and adhered to the Strengthening the Reporting of Observational studies in Epidemiology (STROBE) guidelines. The requirement of written informed consent was waived because the data were de-identified.

### Study design and participants

This retrospective observational cohort study was conducted at the People’s Hospital of Jianyang and the Affiliated Hospital of Southwest Medical University. It analyzed patients who underwent 24-hour Holter from January 1st, 2021 to January 1st, 2022 in six surgical departments of Orthopedics, spine surgery, urology surgery, thoracic surgery, hepatobiliary surgery, and gastrointestinal surgery.

### Inclusion and exclusion criteria

Inclusion criteria: 1) Patients undergoing surgery under general anesthesia. 2) Age ≥18 years old. Exclusion criteria: 3) Absence of 24-hour Holter ECG data. 4) Patients undergoing a second operation. 5) Emergency operation patients.

### 24-hour dynamic electrocardiogram examination and HRV analysis

Non-invasive portable 12-lead Holter monitors were used to record preoperative 24-hour dynamic ECG data. During the recording process, encourage patients to maintain normal daily activities and avoid vigorous exercise. The data were collected by a Hangzhou Baihui ECG workstation with a collection speed of 10 KHz. All non-sinus pulsations and artifacts were excluded in the HRV analysis. ECG physicians performed data analysis at the Affiliated Hospital of Southwest Medical University. Low HRV was diagnosed when the standard deviation (SDNN) of the RR interval of all sinus heartbeats was <100 ms [9, 10]. Patients were divided into low and high HRV groups.

### Data collection

Data from the study were extracted on July 20th, 2023. De-identification technology was used to process and analyze the data, to ensure the protection of patient information. ①Basic information was collected according to the electronic medical record system of Donghua Medical: patient admission number, name, age, sex, department, ASA grade, preoperative comorbidities (including hypertension, coronary heart disease, diabetes, cerebral infarction, chronic lung disease, thrombosis). ②Collection of 24-hour Holter ECG abnormal data: According to the reported results, ECG abnormal results were divided into 10 categories, including sinus arrhythmia, Ectopic arrhythmia, Atrioventricular block, Conduction block disease, ST-T changes, T-wave change, Electrical axis deviation, Prolonged QT interval, Pathological Q wave, Type B ventricular preexcitation.③ Postoperative outcomes: occurrence of PND recorded in psychiatric consultation and discharge diagnosis, in-hospital death, postoperative transfer to ICU, length of stay in the hospital medical record system. Among them, the occurrence of PND is according to ICD-9/10 diagnostic code 10 of the Ninth or Tenth Revision of the International Classification of Diseases. Patients diagnosed with delirium, mild cognitive impairment, and dementia during hospitalization are defined as having PND, and the corresponding diagnostic code is shown in S1 Table.

### Statistical analysis

SPSS 26.0 software was used for statistical analysis. In univariate analysis, the measurement data of non-normal distribution (age and length of stay) were expressed by Median [25th– 75th percentile]. The comparison between groups was conducted by nonparametric test: Kruskal–Wallis rank sum test or Wilcoxon rank sum test. The counting data (such as the number of abnormal results of various dynamic electrocardiograms, postoperative ICU transfer, etc.) were expressed as percentages (%). The chi-square test or Fisher exact probability method was used to compare groups. Univariate analysis was used to screen possible perioperative adverse outcome risk factors, and integrated the independent variables with a significance level of P < 0.1 into a multivariate logistic regression analysis. The variables of the univariate analysis of PND occurrence were incorporated into the multivariate logistic stepwise positive regression model, and the PND prediction model was constructed.

The PND prediction model nomogram diagram was constructed by R 4.0.3 statistical software. The calibration curve was drawn by bootstrap resampling 1000 times for internal verification to evaluate the prediction ability of nomogram. The discriminant power and goodness of fit of the model were tested by the Hosmer–Lemeshow test and receiver operating characteristic (ROC) curve.

Subgroup analyses were carried out to evaluate the robustness of the results. To assess whether the preoperative relationship between HRV and PND was changed by other variables, we performed a subgroup analysis, including age (< 65 years and ≥ 65 years), gender (female and male), ASA (II and III), and types of surgery (gastrointestinal, hepatobiliary, spinal, orthopedic, urinary) to assess the association between outcomes and exposures among different subgroups.

The difference was statistically significant (P < 0.05).

## Results

### Demographic, clinical characteristics of non-cardiac surgery patients

**Fig 1:**
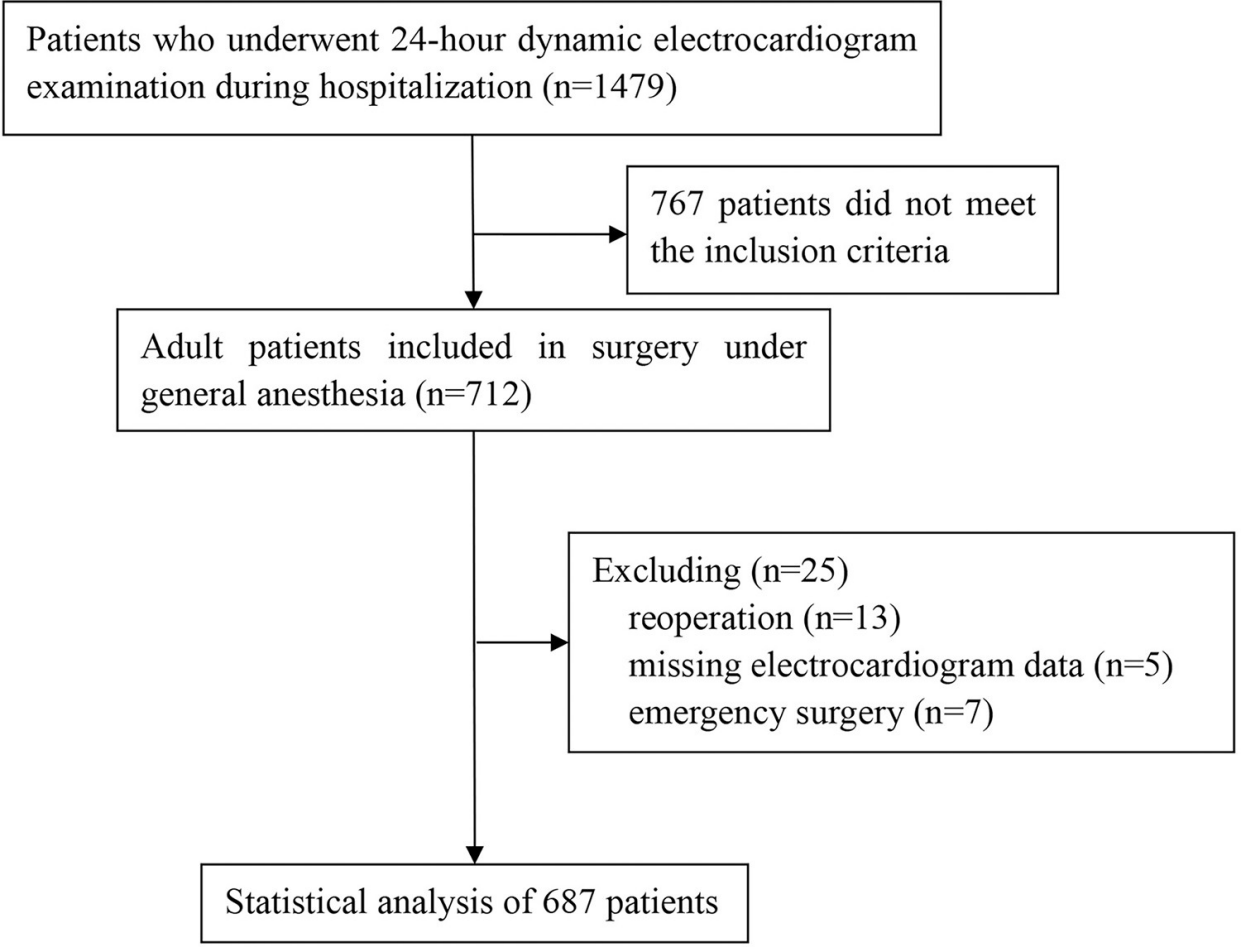
Study flow chart.

A total of 712 patients were enrolled, including 13 who underwent reoperation, 5 with missing original ECG data, and 7 who underwent emergency surgery. Finally, 687 patients were included in this study for statistical analysis. The flow chart is shown in Fig 1. The incidence of low HRV and dope PND were 36.7% and 7.6%, respectively. There was no significant difference in department distribution and sex between the two groups (P >0.05), but it was statistically significant concerning the age and ASA grade difference (P <0.001). In addition, the age of patients in the lower HRV group [68 (58–74)] was older than that in the normal HRV group [70 (64–76)]. There was a significant difference in preoperative thrombosis, diabetes, and cerebral infarction between the two groups in the HRV reduction and the HRV normal groups. Still, there was no significant difference in other complications between the two groups (P >0.05) (Table 1).

**Table 1:** Characteristics and variable distribution of the two groups.

	HHRV(n = 442)	LHRV (n = 245)	P-value
Department			0.298
Gastrointestinal	229 (51.8)	126 (51.4)	
Hepatological	40 (9.0)	22 (9.0)	
Urology	58 (13.1)	23 (9.4)	
Spinal	17 (3.8)	13 (5.3)	
Orthopaedics	56 (12.7)	43 (17.6)	
Thoracic	42 (9.5)	18 (7.3)	
Gender			0.692
Female	170 (38.5)	98(40.0)	
Male	272 (61.5)	147(60.0)	
Age (years)	68 [58–74]	70[64–76]	0.001
ASA grade			0.000
Ⅱ	265 (60)	111 (45.3)	
Ⅲ	177 (40)	134 (54.7)	
Complication			
Thrombus	15 (3.4)	18 (7.3)	0.020
Chronic lung disease	17 (3.8)	15 (6.1)	0.175
Diabetes	37 (8.4)	34 (13.9)	0.023
Hypertension	147 (33.3)	96 (39.2)	0.120
CHD	32 (7.2)	17 (6.9)	0.883
Cerebral infarction	13 (2.9)	24 (9.8)	0.000
Lacunar infarction	16 (3.6)	9 (3.7)	0.971

Data are expressed as median [IQR] or n (%).

Abbreviations: IQR, interquartile range; HRV, heart rate variability; LHRV, low heart rate variability; HHRV, high heart rate variability; CHD, coronary artery heart disease.

### Difference of abnormal constituent ratio of ECG

The incidence of sinus arrhythmias and T wave changes in patients with decreased HRV was significantly lower than those with normal HRV. There was no significant difference in other ECG abnormalities between the two groups (Table 2).

**Table 2:** Abnormal ECG of the two groups.

	HHRV (n = 442)	LHRV (n = 245)	P-value
Sinus arrhythmias	67 (15.2)	15 (6.1)	<0.001
Ectopic arrhythmias	439 (99.3)	243 (99.3)	1.000
AVB	15 (3.4)	7 (2.9)	0.702
Conduction block	45 (10.2)	16 (6.5)	0.107
ST-T changes	102 (23.1)	68 (27.8)	0.173
T wave changes	61 (13.8)	17 (6.9)	0.007
Axis shift	5 (1.1)	1 (0.4)	0.430
Prolonged QT	0 (0.0)	1 (0.4)	0.357
Pathological Q wave	0 (0.0)	1 (0.4)	0.357
WPW	2 (0.5)	0 (0.0)	0.540

Data are expressed as n (%).

Abbreviations: AVB, atrioventricular block; WPW, Wolf–Parkinson–White; LHRV, low heart rate variability; HHRV, high heart rate variability

### Perioperative outcome and hospitalization of patients with different HRV values

Among all the patients, there was no hospital death; 80 (11.6%) were transferred to ICU for observation, and 52 (7.6%) developed PND during hospitalization. The postoperative transfer rate to ICU in the HRV reduction group was significantly higher than in the HRV normal group (P = 0.09). The incidence of PND during hospitalization in the HRV decreased group was significantly higher than in the HRV normal group (P = 0.002). There was a significant difference in hospital stay between the two groups (P <0.05). The hospitalization time of patients with lower HRV was longer than those with normal HRV (Table 3).

**Table 3:** Perioperative outcomes of the two groups.

	HHRV (n = 442)	LHRV (n = 245)	P-value
PND	23 (5.2)	29 (11.8)	0.002
Transferred to the ICU	41 (9.3)	39 (15.9)	0.009
Hospital stays (days)	13 (10,18)	15 (11–19)	0.015

Data are expressed as median [IQR] or n (%).

Abbreviations: IQR, interquartile range; LHRV, low heart rate variability; HHRV, high heart rate variability; PND, perioperative neurological disease; ICU, intensive care unit

### Multivariate logistic regression analysis of influencing factors of PND

Univariate analysis showed that age, HRV, chronic lung disease, lacunar cerebral infarction, sinus tachycardia, frequent atrial premature beats, and ST-T segment changes were associated with PND (P<0.1) (S2 Table). After adjusting the confounding factors, we found that the decrease in HRV was an independent risk factor for PND. The HRV decrease is related to the increased risk of PND [(Adjust odds ratio OR = 2.095, 95% confidence interval CI: 1.160–3.784; P = 0.014]. Age is also an independent risk factor for PND (Table 4).

**Table 4:** Multivariate logistic regression analysis of influencing factors of PND.

	Adjust OR	95%CI	P-value
Age (years)	1.043	1.010–1.077	0.010
HRV	2.095	1.160–3.785	0.014
Chronic lung disease	2.225	0.831–5.956	0.111
Lacunar infarction	2.174	0.709–6.667	0.174
ST	2.542	0.370–17.479	0.343
Frequent APB	1.393	0.744–2.609	0.300
ST-T changes	1.404	0.747–2.640	0.292

Data are expressed as n (%).

Abbreviations: ST, sinus tachycardia; APB, atrial premature beats; OR, odds ratio; CI, Confidence interval

### Multivariate logistic regression analysis predictive model establishment, and evaluation of PND in patients undergoing non-cardiac surgery

**Fig 2:**
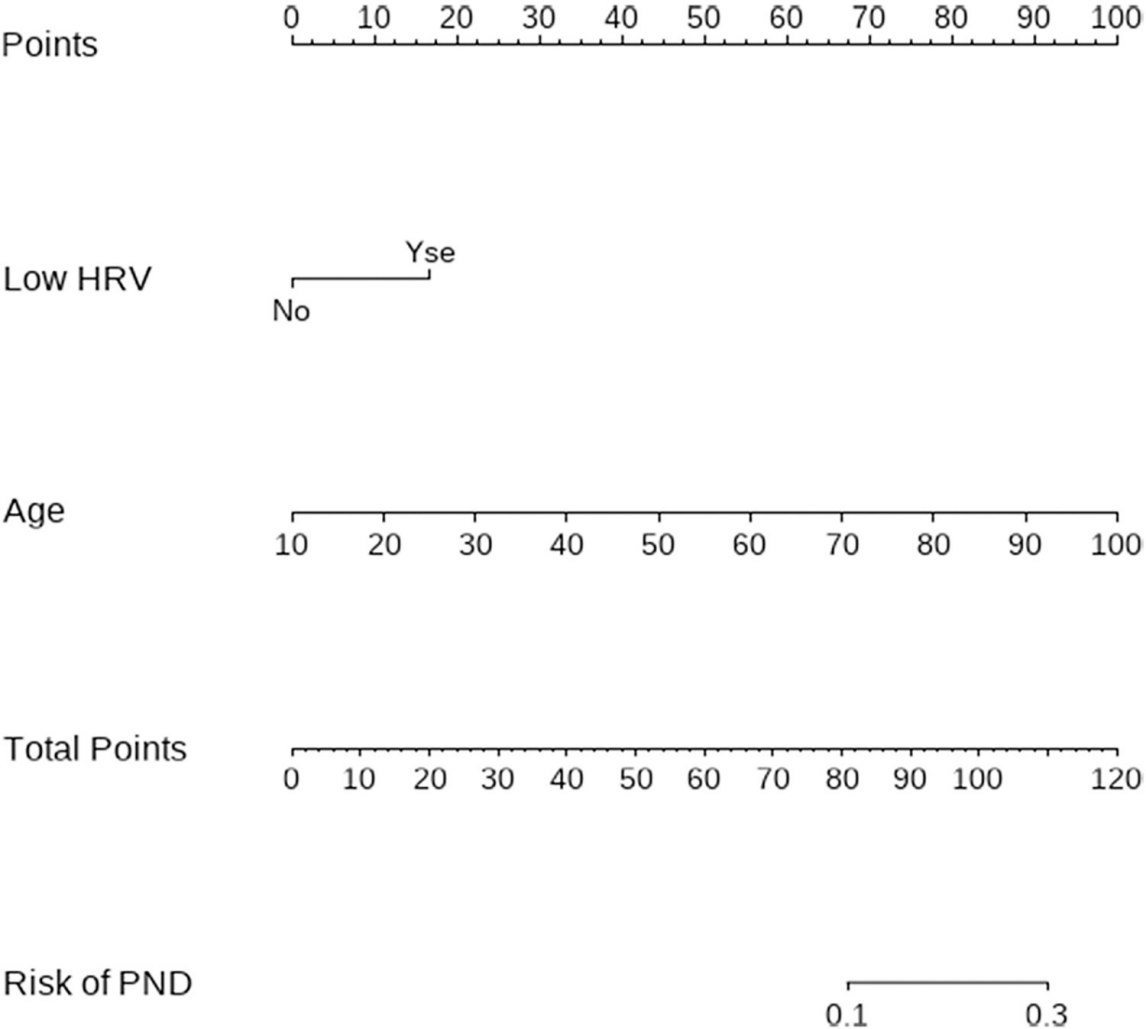
The PND risk nomogram line plots.

**Fig 3:**
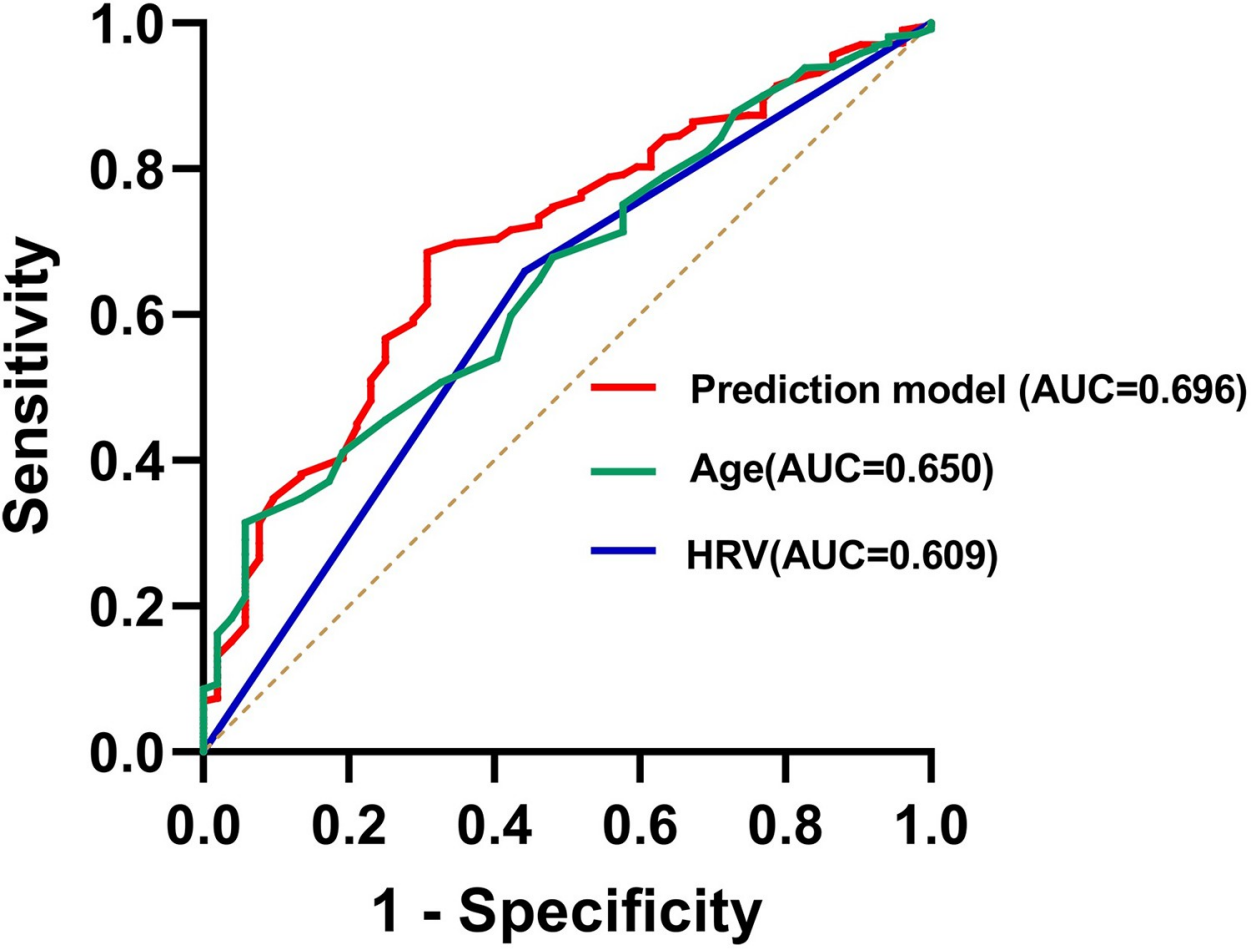
The ROC curve presents the predictive performance of age, HRV, and the prediction model for the risk of PND established through the results of multi-factor logistic regression analysis for the occurrence of PND.

A decrease in HRV and age were independent risk factors for PND (Table 4). The equation for predicting the risk of PND in patients undergoing non-cardiac surgery based on multi-factor logistic regression was Logit(P)=−6.450+0.015×age+0.772×HRV(decreased=1,normal=0). The PND risk nomogram line plots of the model obtained by multi-factor logistic regression are shown in Fig 2. The Hosmer–Lemeshow test was used to detect the goodness of fit for the regression equation (P = 0.781). Based on the outcomes of the multi-factor logistic regression analysis, the prediction model had an AUC of 0.696 (95% CI: 0.626–0.766, P <0.001), indicating its capacity to forecast the occurrence of PND (Fig 3).

### Subgroup analyses

**Fig 4:**
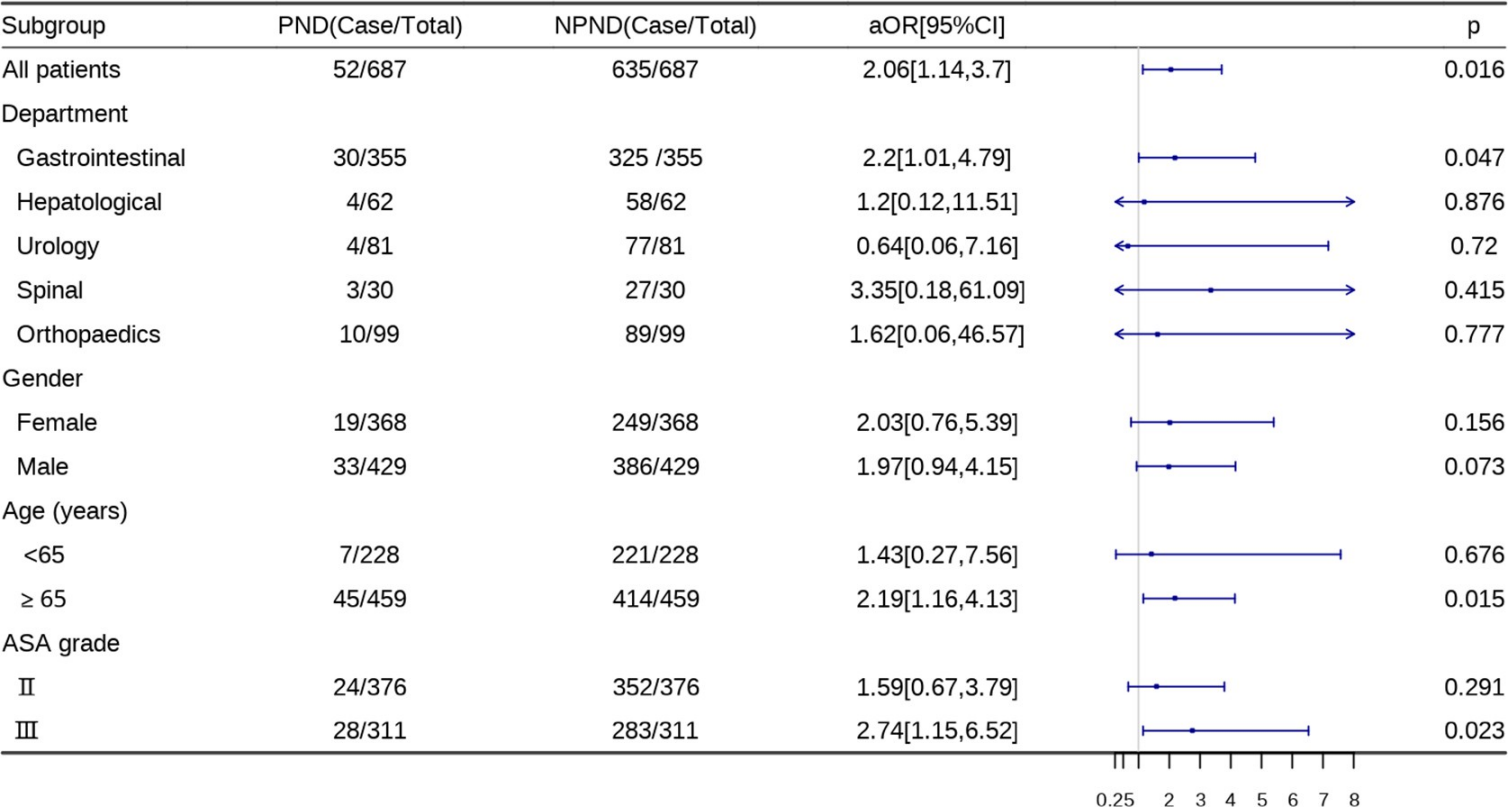
The relationship between low HRV and PND after model adjustment.

In the subgroup analysis, the association between HRV and PND occurrence risk was generally consistent in most subgroups and did not change significantly after additional adjustments. It is worth noting that there was a significant correlation between preoperative low HRV and PDN in patients ≥ 65 years old, gastrointestinal surgery and ASA grade Ⅲ subgroup(P<0.05) (Fig 4).

## Discussion

This study found that low HRV was an independent risk factor for PND. In addition, patients with lower HRV displayed a higher ICU transfer rate, longer hospital stay, and greater impact on the perioperative outcome than patients with normal HRV, which aligns with the results of studies conducted outside China [7, 8, 11]. HRV is the fluctuation of the time interval between adjacent R waves on ECG [12]. It can reflect the change of difference in successive heartbeat cycles, a reliable and non-invasive method for detecting fluctuations in the autonomic nervous system (ANS) [13]. Low HRV is associated with a poor prognosis in many diseases [5, 14, 15] and can also reflect the state of the brain [16]. It is a powerful predictor for cognitive decline, with hypotheses of the related mechanism including brain-mandrel [17, 18] and circulatory disorders that lead to cerebral hypoperfusion [19].

PND is a common perioperative neurological complication. The reported incidence of PND varies greatly, depending on the definition of PND, study population, type of surgery, evaluation time, and cognitive test methods [20]. Patients with PND are characterized by inattention, decreased learning ability, and impaired memory, which affects postoperative functional recovery and prognosis, prolong hospital stay, and may lead to depression and suicidal behavior in severe cases [21, 22]. However, effective treatments for PND are currently unavailable. It is particularly important to predict the occurrence of PND in advance and implement relevant preventive measures to improve the prognosis of surgical patients [23]. The PND prediction index is a popular research topic, and advanced age is a known risk factor for PND [24]. The brains of older patients are vulnerable to anesthesia and surgery due to mechanisms including age-related chronic neuroinflammation, nerve cell death, and dysfunction of the autonomic nervous system [25–27]. Our investigation revealed a significantly higher incidence of perioperative adverse outcomes in older patients than in young patients. Age was found to be an independent risk factor for PND, postoperative transfer to ICU, and prolonged hospital stay. We also found that a decrease in HRV was independently related to the occurrence of PND. HRV, as a common evaluation index of ANS function, has verified that ANS dysfunction is a potential mechanism of PND. Low HRV can be a predictor of PND in clinical practice.

The prevalence of ventricular premature and frequent ventricular premature was 67.7% and 7.7%, respectively, in a 24-hour DCG of outpatients with palpitation in China. The LF/HF ratio of patients with frequent ventricular premature increased, indicating that low HRV is related to the occurrence of arrhythmia [28]. This study found that the prevalence of sporadic and frequent VPB was 57.6% and 23.9%, respectively, different from those reported in the abovementioned studies. The main reason for this is that the objects of the studies were different.

This study found that coronary heart disease, AF, changes in the ST-T segment, and low HRV were independent risk factors for postoperative ICU transfer, which was consistent with the results of previous studies [7, 8, 11]. Some abnormal ECG results can predict the difficulty in managing perioperative anesthesia, and patients need to be transferred to the ICU for close monitoring after surgery. However, this needs to be confirmed in subsequent prospective studies.

Previous studies have shown that the clinical value of preoperative ordinary ECG examination is limited [29]. A 24-hour DCG can better reflect patients’ circadian rhythm and occasional severe ECG changes [30] and comprehensively analyze HRV occurring day and night. As a marker to identify the balance between sympathetic and parasympathetic tension, HRV can predict disease mortality, sudden death, risks of cardiovascular diseases, and other morbidities [31]. However, the clinical application of HRV is not common due to the following reasons: HRV analysis requires sinus rhythm; long-term ECG recording is needed to analyze HRV during the day and night; and the best analysis method has not been determined, including the time domain index, frequency domain index, heart rate disorder analysis index, geometric analysis index, and a series of nonlinear analysis indexes, each of which reflects different aspects of HRV and has a specific correlation with some diseases. At present, the detection conditions of HRV are limited, and research on the mechanism is not mature. However, with the standardization of measurement methods and further clarification of the related mechanism, the analysis of HRV can provide more help for clinical practice.

This study had limitations. First, this was a single-center, retrospective study, which limits the wider applicability of our research results, and introduces confounding factors. Second, the diagnosis of PND was not comprehensive. This study has missed diagnoses regarding the application of commonly used scales in diagnosing PND, resulting in a low incidence of PND; thus, further prospective studies must be conducted. The advantage of this study is that the sample size is large, and it is about the long-term record of HRV.

In conclusion, various 24-hour DCG abnormalities were associated with adverse outcomes in non-cardiac surgery patients. Low HRV was independently related to PND and postoperative ICU transfers. In our subsequent studies, we plan to investigate the relationship between various HRV indices and PND, which will aid in the risk stratification of high-risk patients with PND. In addition, further studies on the mechanism behind the low HRV and the occurrence of PND should be conducted to provide new research directions and specific therapeutic targets for clinical prevention and PND treatment.

## Supporting information

S1 Table PND diagnostic code corresponding to ICD-9/10. (DOCX)

S2 Table PND univariate analysis. (DOCX)

## References

[1] Oresanya LB, Lyons WL, Finlayson E. Preoperative assessment of the older patient: a narrative review. Jama. 2014;311(20):2110–20. Epub 2014/05/29. doi: 10.1001/jama.2014.4573 . 24867014

[2] Griffiths R, Alper J, Beckingsale A, Goldhill D, Heyburn G, Holloway J, et al. Management of proximal femoral fractures 2011: Association of Anaesthetists of Great Britain and Ireland. Anaesthesia. 2012;67(1):85–98. doi: 10.1111/j.1365-2044.2011.06957.x . 22150501

[3] Marcus G, Zilberstein A, Kumetz I, Love I, Mengesha B, Tsiporin F, et al. ECG changes after non-cardiac surgery: a prospective observational study in intermediate-high risk patients. Minerva Anestesiol. 2021;87(3):283–93. doi: 10.23736/S0375-9393.20.14697-2 . 33325213

[4] GhiculeteDaniela J S, G. LP, HongAaron FarcasMonica, BarrettKeith, et al. Routine Preoperative Electrocardiograms in Patients at Low Risk for Cardiac Complications During Shockwave Lithotripsy: Are They Useful? J Endourol. 2019;33(4):251–8. 10.1089/end.2019.0053 30724110

[5] Thayer J, Yamamoto S, Brosschot J. The relationship of autonomic imbalance, heart rate variability and cardiovascular disease risk factors. Int J Cardiol. 2010;141(2):122–31. doi: 10.1016/j.ijcard.2009.09.543 . 19910061

[6] Liu K, Elliott T, Knowles M, Howard R. Heart rate variability in relation to cognition and behavior in neurodegenerative diseases: A systematic review and meta-analysis. Ageing Res Rev. 2022;73:101539. doi: 10.1016/j.arr.2021.101539 . 34883203 PMC8783051

[7] Tsukakoshi D, Yamamoto S, Nojima I, Sato M, Furuhashi K, Takeda S, et al. Association between postoperative delirium and heart rate variability in the intensive care unit and readmissions and mortality in elderly patients with cardiovascular surgery. Heart Vessels. 2022:438–47. doi: 10.1007/s00380-022-02173-1 . 36205773

[8] Echizen M, Satomoto M, Miyajima M, Adachi Y, Matsushima E. Preoperative heart rate variability analysis is as a potential simple and easy measure for predicting perioperative delirium in esophageal surgery. Ann Med Surg (Lond). 2021;70:102856. doi: 10.1016/j.amsu.2021.102856 . 34584685 PMC8452778

[9] Hilgarter K, Schmid-Zalaudek K, Csanády-Leitner R, Mörtl M, Rössler A, Lackner HK. Phasic heart rate variability and the association with cognitive performance: A cross-sectional study in a healthy population setting. PloS one. 2021;16(3):e0246968. Epub 2021/03/02. doi: 10.1371/journal.pone.0246968 ; PubMed Central PMCID: PMC7920382. 33647023 PMC7920382

[10] Heart rate variability. Standards of measurement, physiological interpretation, and clinical use. Task Force of the European Society of Cardiology and the North American Society of Pacing and Electrophysiology. European heart journal. 1996;17(3):354–81. Epub 1996/03/01. . 8737210

[11] Sun J, Zhang Q, Lin B, He M, Pang Y, Liang Q, et al. Association Between Postoperative Long-Term Heart Rate Variability and Postoperative Delirium in Elderly Patients Undergoing Orthopedic Surgery: A Prospective Cohort Study. Front Aging Neurosci. 2021;13:646253. doi: 10.3389/fnagi.2021.646253 . 34135747 PMC8200544

[12] Shaffer F, Ginsberg J. An Overview of Heart Rate Variability Metrics and Norms. Frontiers in public health. 2017;5:258. doi: 10.3389/fpubh.2017.00258 . 29034226 PMC5624990

[13] Evangelista G, Dono F, Consoli S, Lanzone J, Corniello C, Russo M, et al. Heart rate variability modification as a predictive factor of sudden unexpected death in epilepsy: How far are we? A systematic review and meta-analysis. European journal of neurology. 2023. Epub 2023/03/19. doi: 10.1111/ene.15792 . 36932903

[14] Chang YM, Huang YT, Chen IL, Yang CL, Leu SC, Su HL, et al. Heart rate variability as an independent predictor for 8-year mortality among chronic hemodialysis patients. Scientific reports. 2020;10(1):881. Epub 2020/01/23. doi: 10.1038/s41598-020-57792-3 ; PubMed Central PMCID: PMC6972735. 31964940 PMC6972735

[15] Tsuji H, Venditti FJ Jr., Manders ES, Evans JC, Larson MG, Feldman CL, et al. Reduced heart rate variability and mortality risk in an elderly cohort. The Framingham Heart Study. Circulation. 1994;90(2):878–83. Epub 1994/08/01. doi: 10.1161/01.cir.90.2.878 . 8044959

[16] Forte G, Favieri F, Casagrande M. Heart Rate Variability and Cognitive Function: A Systematic Review. Front Neurosci. 2019;13:710. doi: 10.3389/fnins.2019.00710 . 31354419 PMC6637318

[17] Silvani A, Calandra-Buonaura G, Dampney R, Cortelli P. Brain-heart interactions: physiology and clinical implications. Philos Trans A Math Phys Eng Sci. 2016;374(2067). doi: 10.1098/rsta.2015.0181 . 27044998

[18] DXZDZGD X. The effect of baicalin on cognitive function of cerebral ischemia-reperfusion injury in mice through PGE2. The Journal of Practical Medicine. 2023;39(15):1881–7. doi: 10.3969/j.issn.1006-5725.2023.15.005

[19] Jia P, Lee H, Chan J, Yiu K, Tsoi K. Long-Term Blood Pressure Variability Increases Risks of Dementia and Cognitive Decline: A Meta-Analysis of Longitudinal Studies. Hypertension. 2021;78(4):996–1004. doi: 10.1161/HYPERTENSIONAHA.121.17788 . 34397274

[20] Ju LS, Morey TE, Seubert CN, Martynyuk AE. Intergenerational Perioperative Neurocognitive Disorder. Biology. 2023;12(4). Epub 2023/04/28. doi: 10.3390/biology12040567 ; PubMed Central PMCID: PMC10135810. 37106766 PMC10135810

[21] Liu B, Huang D, Guo Y, Sun X, Chen C, Zhai X, et al. Recent advances and perspectives of postoperative neurological disorders in the elderly surgical patients. CNS Neurosci Ther. 2022;28(4):470–83. doi: 10.1111/cns.13763 . 34862758 PMC8928923

[22] Jia L, Du Y, Chu L, Zhang Z, Li F, Lyu D, et al. Prevalence, risk factors, and management of dementia and mild cognitive impairment in adults aged 60 years or older in China: a cross-sectional study. The Lancet Public health. 2020;5(12):e661–e71. Epub 2020/12/04. doi: 10.1016/S2468-2667(20)30185-7 . 33271079

[23] Feng L, Wang Y, Zeng D, Wang M, Duan X. Predictors of cognitive decline in older individuals without dementia: An updated meta-analysis. Annals of clinical and translational neurology. 2023;10(4):497–506. Epub 2023/01/28. doi: 10.1002/acn3.51740 ; PubMed Central PMCID: PMC10109353 to the manuscript. 36705073 PMC10109353

[24] Xiao Q, Liu Q, Deng R, Gao Z, Zhang Y. Postoperative cognitive dysfunction in elderly patients undergoing hip arthroplasty. Psychogeriatrics. 2020;20(4):501–9. doi: 10.1111/psyg.12516 . 31976614

[25] H OB, Mohan H, Hare CO, Reynolds JV, Kenny RA. Mind Over Matter? The Hidden Epidemic of Cognitive Dysfunction in the Older Surgical Patient. Annals of surgery. 2017;265(4):677–91. Epub 2016/08/19. doi: 10.1097/SLA.0000000000001900 . 27537541

[26] Jiang Y, Yabluchanskiy A, Deng J, Amil FA, Po SS, Dasari TW. The role of age-associated autonomic dysfunction in inflammation and endothelial dysfunction. GeroScience. 2022;44(6):2655–70. Epub 2022/07/01. doi: 10.1007/s11357-022-00616-1 ; PubMed Central PMCID: PMC9768093. 35773441 PMC9768093

[27] Soreq L, Rose J, Soreq E, Hardy J, Trabzuni D, Cookson MR, et al. Major Shifts in Glial Regional Identity Are a Transcriptional Hallmark of Human Brain Aging. Cell reports. 2017;18(2):557–70. Epub 2017/01/12. doi: 10.1016/j.celrep.2016.12.011 ; PubMed Central PMCID: PMC5263238. 28076797 PMC5263238

[28] Dong Y, Li X, Zheng W, Man Y, Liu J, Yu P, et al. Prevalence and heart rate variability characteristics of premature ventricular contractions detected by 24-hour Holter among outpatients with palpitations in China: a cross-sectional study. BMJ open. 2022;12(8):e059337. doi: 10.1136/bmjopen-2021-059337 . 35918118 PMC9351320

[29] Studzińska D, Polok K, Rewerska B, Kotynia M, Rewerski P, Włudarczyk A, et al. Prognostic value of preoperative electrocardiography in predicting myocardial injury after vascular surgery. Kardiologia polska. 2022. doi: 10.33963/KP.a2022.0085 . 35344585

[30] Dalise AM, Prestano R, Fasano R, Gambardella A, Barbieri M, Rizzo MR. Autonomic Nervous System and Cognitive Impairment in Older Patients: Evidence From Long-Term Heart Rate Variability in Real-Life Setting. Front Aging Neurosci. 2020;12:40. Epub 2020/03/29. doi: 10.3389/fnagi.2020.00040 ; PubMed Central PMCID: PMC7079686. 32218729 PMC7079686

[31] Sessa F, Anna V, Messina G, Cibelli G, Monda V, Marsala G, et al. Heart rate variability as predictive factor for sudden cardiac death. Aging. 2018;10(2):166–77. Epub 2018/02/25. doi: 10.18632/aging.101386 ; PubMed Central PMCID: PMC5842851. 29476045 PMC5842851

