# Supplementary material for: Correlation between heart rate variability and perioperative neurocognitive disorders in patients undergoing non-cardiac surgery: A retrospective cohort study
Source: PLoS One. 2024 Apr 2;19(4):e0297337. doi: 10.1371/journal.pone.0297337 (PMC10986934; doi:10.1371/journal.pone.0297337)
Supplement: S1 Table — (DOCX) [file pone.0297337.s001.docx]

**S1 Table. PND diagnostic code corresponding to ICD-9/10**

| ICD-9 | ICD-10 |
| --- | --- |
| Delirium |  |
| 289.9, 290.0, 290.11, 290.3, 290.41, 291.0, 293.0, 293.1, 293.89 | R41.0, F03.90, F03.91 |
| Mild cognitive impairment |  |
| 331.83 | G31.84 |
| Dementia |  |
| 290.0, 290.11, 290.3, 290.41 | F03.90, F03.91 |

Abbreviations: ICD, International Statistical Classification of Diseases and Related Health Problems; PND, Perioperative neurological disease
