# Supplementary material for: Correlation between heart rate variability and perioperative neurocognitive disorders in patients undergoing non-cardiac surgery: A retrospective cohort study
Source: PLoS One. 2024 Apr 2;19(4):e0297337. doi: 10.1371/journal.pone.0297337 (PMC10986934; doi:10.1371/journal.pone.0297337)
Supplement: S2 Table — (DOCX) [file pone.0297337.s002.docx]

**S2 Table. PND univariate analysis**

|  | PND | NPND | P-value |
| --- | --- | --- | --- |
| Age (years) | 73(67.25,79) | 68(60,74) | 0.000 |
| Gender |  |  | 0.704 |
| Female | 33（7.9） | 386(92.1) |  |
| Male | 19（7.1） | 33(7.9) |  |
| HRV |  |  | 0.002 |
| decreased | 29(11.8) | 216(88.2) |  |
| normal  ASA grade  Ⅱ  Ⅲ  Complication | 23(5.2)  352(55.4)  283(44.6) | 419(94.8)  24(46.2)  28(53.8) | 0.196 |
| Thrombus | 1(3) | 32（97） | 0.501 |
| Chronic lung disease | 6(2.4） | 26(81.3) | 0.035 |
| Diabetes | 7(9.9) | 64(90.1) | 0.441 |
| Hypertension | 23(18.4) | 220(90.5) | 0.165 |
| CHD | 2(3.7) | 47(95.9) | 0.495 |
| Cerebral infarction | 6(2.8) | 31(83.8) | 0.085 |
| Lacunar infarction | 5(20) | 20(80) | 0.045 |
| Sinus arrhythmias | 4(6.2） | 78(95.1) | 0.326 |
| ST | 2(28.6) | 5(71.4) | 0.092 |
| SB | 1(2.4) | 40(97.6) | 0.329 |
| Sinus arrest | 0(0.0) | 3(100) | 1.000 |
| Sinus arrhythmia | 0(0） | 0(0) |  |
| SSS | 1(2.3) | 42(39.7) | 0.296 |
| Ectopic arrhythmias | 52(7.6) | 630(92.4) | 1.000 |
| Junctional ectopic beats | 1(1.6) | 20(95.2) | 0.940 |
| VEB | 1(20) | 4(80) | 0.326 |
| Occasional APB | 28(6.7) | 392(93.3) | 0.262 |
| Occasional VPB | 28(30) | 368(92.9) | 0.564 |
| Frequent APB | 24(6) | 196(89.1) | 0.023 |
| Frequent VPB | 14(8.5) | 150(91.5) | 0.591 |
| VT | 0(0) | 6(100) | 1.000 |
| Ventricular flutter | 0(0) | 3(100) | 1.000 |
| AF | 2(5.7) | 33(94.3) | 0.922 |
| VF | 0 | 0 |  |
| AVB | 2(9.1) | 20(90.9) | 1.000 |
| First-degree | 2(20) | 8(80) | 0.171 |
| Second degree Ⅰ | 0(0) | 9(100) | 1.000 |
| Second degree Ⅱ | 0(0) | 4(100) | 1.000 |
| Third degree | 0 | 0 |  |
| Conduction block | 4(6.6) | 57(93.4) | 0.953 |
| RBBB | 4(8.3) | 44(91.7) | 1.000 |
| LBBB | 0(0) | 8(100) | 1.000 |
| Intraventricular block | 0(0) | 2(100) | 1.000 |
| LAH | 0(0) | 6(100) | 1.000 |
| ST-T changes | 18(10.6) | 152(89.4) | 0.086 |
| ST segment depression | 0(0) | 7(100) | 1.000 |
| ST segment elevation | 0(0) | 2(100) | 1.000 |
| ST-T segment change | 18(11) | 145(89) | 0.055 |
| T wave changes | 4(5.1) | 74(94.9) | 0.387 |
| T wave inversion | 1(33.3%) | 2(66.7) | 0.211 |
| Flat T wave | 0 | 0 |  |
| T wave change | 3(4) | 72(96) | 0.216 |
| Axis shift | 0(0) | 6(100) | 1.000 |
| RAD | 0(0) | 1(100) | 1.000 |
| LAD | 0 | 0 |  |
| LVHV | 0(0) | 5(100) | 1.000 |
| Prolonged QT | 0(0) | 1(100) | 1.000 |
| Pathological Q wave | 0(0) | 1(100） | 1.000 |
| WPW | 0(0) | 2(100) | 1.000 |

Data are expressed as median [IQR] or n (%).

IQR, interquartile range; PND, Perioperative neurological disease ；NPND, Not Perioperative neurological disease HRV, heart rate variability; CHD, coronary artery heart disease; ST, sinus tachycardia; SB, sinus bradycardia; SSS, sick sinus syndrome; VEB, ventricular escape beats; APB, atrial premature beats; VPB, ventricular premature beats.

VT, ventricular tachycardia; AF, atrial fibrillation; VF, ventricular fibrillation; AVB, atrioventricular block; RBBB, right bundle branch block; LBBB, left bundle branch block; LAH, left anterior hemiblock; RAD, right axis deviation; LAD, left axis deviation; LVHV, left ventricular high voltage; WPW, Wolf–Parkinson–White.
